# Vascular reconstruction of segmental intestinal grafts using autologous internal iliac vessels

**DOI:** 10.1093/gastro/goab016

**Published:** 2021-06-21

**Authors:** Guosheng Wu, Yinglun Wu, Mian Wang, Wentong Zhang, Chaoxu Liu, Tingbo Liang

**Affiliations:** 1 Intestinal Transplant Center, The First Affiliated Hospital, Zhejiang University School of Medicine, Hangzhou, P.R. China; 2 Section of Plastic Surgery, Dartmouth-Hitchcock Medical Center, Lebanon, NH, USA; 3 State Key Laboratory of Cancer Biology & National Clinical Research Center for Digestive Diseases, Xijing Hospital, Air Force Military Medical University, Xi'an, China

**Keywords:** internal iliac vessel graft, intestinal autotransplantation, living-related intestinal transplantation

## Abstract

**Background:**

The aim of this study was to assess whether the autologous internal iliac artery and vein could be used as an interpositional graft for vascular reconstruction in segmental intestinal allografts and autografts.

**Methods:**

Thirty-four intestinal transplants (19 living-related allografts and 15 autografts) were conducted in our programs between January 2011 and January 2019. Patient characteristics, type of vascular reconstruction, and post-operative complications were reviewed.

**Results:**

There were 20 males and 14 females with a median age of 35 years. Of 34 grafts, 22 (64.7%) (11 allografts and 11 autografts) were revascularized using the autologous internal iliac artery and vein for reconstruction. Vascular reconstruction on the back table took 21 ± 6 min to complete. Both total operative time and cold ischemia time tended to be longer in the vascular-reconstruction group than in the direct-anastomosis group (530 ± 226 vs 440 ± 116 and 159 ± 49 vs 125 ± 66 min, respectively), but these differences were not significant. The incidence of vascular thrombosis tended to be higher in the direct-anastomosis group than in the vascular-reconstruction group (16.7% vs 0%, *P* = 0.118). At a median follow-up of 36.9 months, no stenosis or pseudoaneurysms developed. In 19 allografts, acute rejection occurred in 4 (21.1%) and chronic rejection occurred in 1 (5.2%).

**Conclusions:**

Our results indicate that the use of an autologous internal iliac interposition graft greatly facilitates intestinal graft implantation and minimizes the risk of vascular complications.

## Introduction

Despite recent advances in the medical and surgical management of intestinal failure, intestinal transplantation (ITx) continues to play an important role [1, 2]. To date, nearly 3,000 intestinal transplants have been performed across the world according to data from the International Intestinal Transplant Registry [3, 4]. An intestinal graft can be transplanted alone or in combination with other organs. However, due to improvement in the management of intestinal failure-associated liver disease, the number of liver-inclusive ITx has steadily decreased over the years and isolated ITx has become the most common type performed [5, 6].

An isolated intestinal allograft can be procured from either cadaveric or living donors with a pedicle containing the superior mesenteric artery (SMA) and superior mesenteric vein (SMV). Due to the short length and small caliber of the donor's mesenteric vessels, it is often technically challenging to connect the donor's mesenteric vessels to the recipient's inferior aorta and vena cava or superior mesenteric vessels and is particularly challenging in living-related ITx (LR-ITx) and pediatric ITx [7, 8]. In these cases, it is necessary to use an interpositional graft for vascular reconstruction.

With advances in organ preservation and surgical techniques, *ex vivo* surgery and autotransplantation have successfully been performed for the kidney, liver, and heart [9–11]. Tzakis *et al*. [12, 13] initially described a novel technique for intestinal autotransplantation (IATx), which involves an *en bloc* removal of a tumor together with the intestine, *ex vivo* tumor resection, followed by reimplantation of the intestinal autograft. We further refined this complex technique with the initial selection and procurement of a healthy segmental bowel autograft, *in vivo* radical tumor resection, followed by intestinal graft reimplantation. In this setting, the use of vascular conduits is frequently required to extend the length of the vascular pedicle of a graft [14–17].

The internal iliac artery and vein may be an ideal option for SMA and SMV reconstruction because they are of adequate length (∼3–4 cm) and diameter for segmental intestinal transplants. In this study, we hypothesized that the use of internal iliac vessels for vascular reconstruction may increase the potential of successful anastomosis and reduce the risk of vascular complications.

## Patients and methods

**Figure 1.**
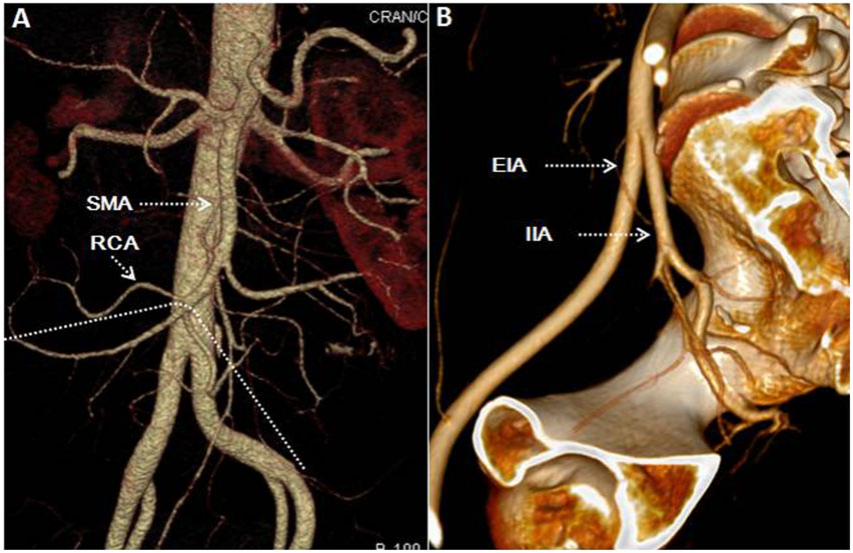
Preoperative 3D reconstruction of CT scan. (A) Anatomy of the superior mesenteric artery (SMA) with the right colic artery (RCA) and planned line of mesenteric resection (dotted line); (B) anatomy of external iliac artery, internal iliac artery (IIA), and its branches.

Thirty-four segmental intestinal transplants, composed of 19 living-related allografts and 15 autografts, were performed at two centers (Digestive Diseases, Xijing Hospital and the First Affiliated Hospital, Zhejiang University School of Medicine) from January 2011 to January 2019. Patients were divided into the direct-anastomosis group and the vascular-reconstruction group. Patient data were retrieved from prospectively maintained computerized databases, flow charts, and medical records. All patients underwent contrast-enhanced computerized tomography (CT) scan as a routine preoperative assessment. CT angiography with 3D image reconstruction was performed to evaluate the donor's intestinal vascular anatomy and the recipient's external and internal iliac arteries (Figure 1). The Institutional Review Board of our hospital approved this procedure (zjswst-2103–110).

## Operative techniques

**Figure 2.**
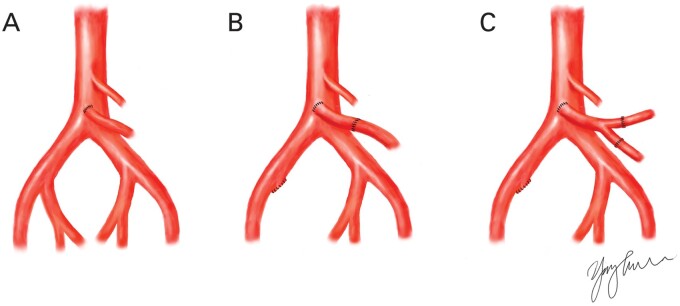
Vascular reconstruction for intestinal transplantation. (A) End-to-side primary anastomosis. (B) Interposed internal iliac graft. (C) Interposition Y-graft using an autologous internal iliac vessel.

**Figure 3.**
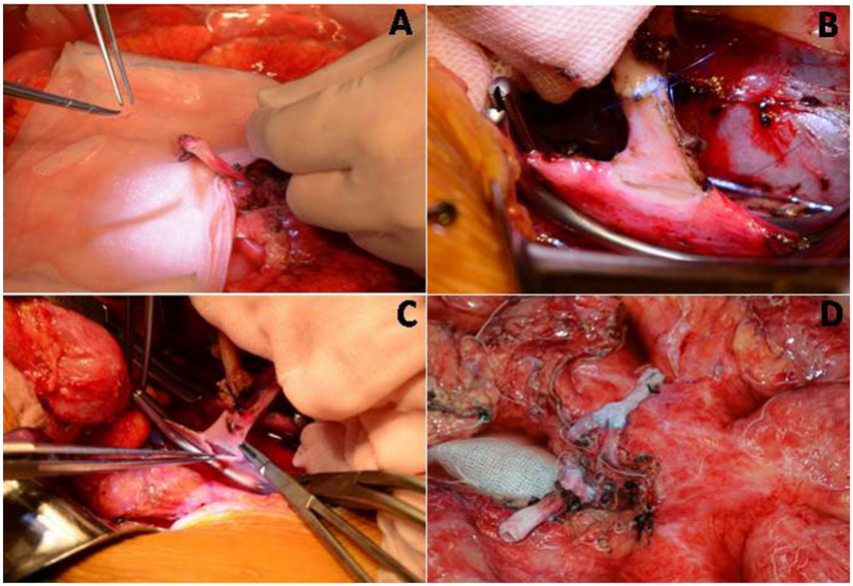
Intraoperative photograph demonstrating vascular reconstruction for intestinal autotransplantation. (A) *Ex vivo* arterial reconstruction. (B) Graft artery anastomosed to the recipient's aorta. (C) Graft vein anastomosed to the recipient's vena cava. (D) Y-graft reconstruction for artery and vein.

The operative techniques of LR-ITx and IATx have previously been described in detail by our team and others [13, 15]. In brief, the operation begins with an upper midline laparotomy incision extending to the suprapubic region. A suitable segment of intestine with reasonable mesenteric vessels for vascular anastomosis is selected and harvested. Once the vessels are transected at the designed line, the graft is removed and immediately flushed through the artery with cold histidine-tryptophan-ketoglutarate solution until clear return from the vein is obtained. Next, the internal iliac vessels on the ipsilateral side of the operator are excised and stumps of the main trunk and important branches are preserved as much as possible. After harvesting, the graft's arterial lumen is flushed with saline containing heparin. Types of vascular reconstruction are illustrated in Figure 2; a back-table reconstruction of the graft's distal SMA and SMV from the autologous internal iliac vessels and intraoperative vascular reconstruction are shown in Figure 3.

Once complete hemostasis is achieved, the bowel autograft is brought into the surgical field and revascularized to the recipient's infrarenal aorta and vena cava or superior mesenteric vessels. The gastrointestinal tract is reconstructed using either an end ileostomy to monitor graft function in LR-ITx or a 45- to 50-cm Roux-en-Y limb for pancreaticoenterostomy, choledochoenterostomy, gastroenterostomy, and ileocolostomy in IATx.

## Post-transplant management

**Figure 4.**
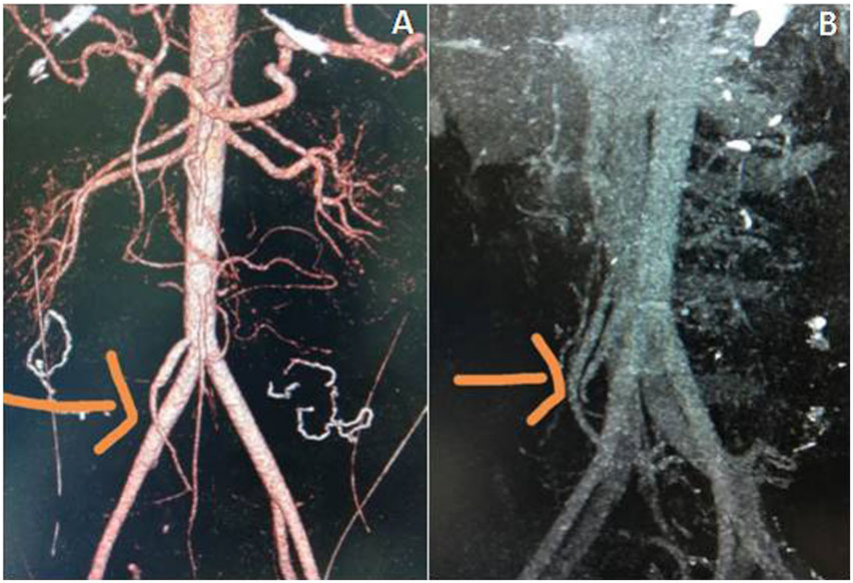
Vascular reconstruction using the autologous iliac artery (A) and vein (B)

After the operation, intravenous albumin is administered to maintain blood levels at >30 g/L. Prophylactic antibiotics are given for 48 hours post-operatively. Vascular complications were closely monitored, including artery or vein thrombosis, stenosis, and pseudoaneurysm. Bedside duplex Doppler sonography is performed to check the patency of the SMA and SMV immediately after patients are transferred to the intensive care unit from the operating room and then daily for 3 consecutive days post-operatively. 3D CT angiography is used to confirm the patency of the graft vessels and the blood supply to the bowel autograft when Doppler imaging is inadequate (Figure 4). Intravenous heparin is initiated on post-operative day 1 to maintain an activated partial thromboplastin time of 1.5‒2.0 times control. Heparin is discontinued within a week and 100 mg of aspirin per day is then maintained for a year. All patients are kept on total parenteral nutrition (TPN) initially. Enteral or oral feeding is resumed within 2 weeks post-operatively and advanced as tolerated. TPN is discontinued gradually as patients are able to take in sufficient enteral nutrition. In the LR-ITx, surveillance endoscopy was performed weekly during the first month, followed by bi-weekly during months 2‒3, once a month during months 4‒6, and every 2 months during months 7‒12 post-operatively.

The primary outcome of this study was vascular complications after the procedure, including vascular thrombosis, arterial stenosis, and pseudoaneurysms etc.

## Statistical analysis

All data are expressed as mean ± standard deviation. Statistical significance was determined using Student’s *t*-test (two-tailed) comparison between two groups of data sets. Fisher exact test was used to determine whether there is a significant difference between the two proportions. A *P*-value of ≤0.05 was considered significant.

## Results

There were 20 men and 14 females with a median age of 35 years. In our series, short gut syndrome was the most common surgical indication for LR-ITx, which is the treatment of choice for patients who develop intractable TPN-related complications such as liver dysfunction, lack of central venous access, and catheter-related sepsis. IATx is reserved for a select subset of patients with locally invasive neoplasm closely involving the SMA without evidence of distant metastases. In this study, these neoplasms commonly originate in the head of the pancreas and the mesenteric root. A more detailed description of these patients was reported in our previous studies [14‒17].

## Operative characteristics

Four types of vascular reconstruction are summarized in Table 1. Of 34 transplants, 6 (17.6%) were directly anastomosed to the remaining native stump of the SMA and SMV without use of an interpositional vascular graft. The remaining 28 (82.4%) were anastomosed to the infrarenal aorta and vena cava. Of these 28 cases, 6 (21.4%) were directly anastomosed without the use of vascular grafts and 22 (78.6%) (11 allografts and 11 autografts) required an iliac interpositional autograft for *ex vivo* vascular reconstruction. Y-graft reconstruction was performed in five cases due to either double vein (*n* = 2) or double arteries (*n* = 3).

**Table 1.** Type of vascular reconstruction

Type	Living-related intestinal transplantation (*n* = 19)	Intestinal autotransplantation (*n* = 15)
I	3	3
II	5	1
III	10	7
IV	1	4

I. End-to-end anastomosis to native remnant superior mesenteric artery and superior mesenteric vein/portal vein without an interpositional graft. II. Direct end-to-side anastomosis to the infrarenal aorta and vena cava without an interpositional graft. III. End-to-side anastomosis with an interposed internal iliac graft. IV. End-to-side anastomosis with an interposition Y-graft reconstruction.

Clinical characteristics of direct anastomosis vs vascular reconstruction are summarized in Table 2. There was no significant difference in age, sex, and type of transplant between the two groups. Vascular reconstruction on the back table took 21 ± 6 min to complete. Both the total operative time and the cold ischemia time tended to be longer in the vascular-reconstruction group than in the direct-anastomosis group (530 ± 226 vs 440 ± 116 and 159 ± 49 vs 125 ± 66 min, respectively), but these differences were not significant.

**Table 2.** Clinical characteristics of patients with direct anastomosis vs vascular reconstruction

Characteristic	Direct anastomosis (type I+II, *n* = 12)	Vascular reconstruction (type III+IV, *n* = 22)	*P*-value
Mean age (years)	32.5 ± 12.8	35.8 ± 11.4	0.445
Female [*n* (%)]	5 (41.6)	9 (40.9)	0.966
Transplantation procedure [*n* (%)]			0.350
Living-related intestinal transplantation	8 (66.7)	11 (50.0)	
Intestinal autotransplantation	4 (33.3)	11 (50.0)	
Mean total operative time (min)	440 ± 116	530 ± 226	0.208
Mean vascular-reconstruction time (min)	–	21 ± 6	
Mean cold ischemia time (min)	125 ± 66	159 ± 49	0.097
Vascular thrombosis [*n* (%)]	2 (16.7%)	0	0.118

## Vascular complications

In our series, 2 of 12 patients in the direct-anastomosis group developed vascular thrombosis after surgery, but there was no thrombosis in patients with vascular reconstruction. The first patient was a 63-year-old man who underwent near-total enterectomy due to a mesenteric desmoid tumor and subsequently received a segmental bowel autograft. We experienced technical difficulties during anastomosis of the graft artery to the recipient's infrarenal aorta due to a short pedicle length, small graft vessel caliber, and severe calcification of the recipient's aorta. The patient developed thrombosis at the SMA anastomosis 48 hours post-operatively. Since the necrotic bowel graft was removed, the patient has been on full TPN support. The second patient received two independent segmental bowel autografts: the graft vein of the first segment was anastomosed to the splenic vein; the graft vein of the second segment with iliac vein Y-graft reconstruction was anastomosed to the infrarenal vena cava. Acute venous thrombus in the first graft was detected at the anastomosis by Doppler ultrasonography 8 hours after IATx. Following immediate open thrombectomy, the graft was successfully salvaged.

The incidence of vascular thrombosis tended to be higher in the direct-anastomosis group than in the vascular-reconstruction group (16.7% vs 0%), but these differences did not reach statistical significance (*P* = 0.118; Table 2). At a median follow-up of 36.9 months (range 3.1‒92.4 months), all vascular anastomoses were widely patent without evidence of arterial stenosis or pseudoaneurysms. In 19 living-related grafts, acute rejection occurred in 4 cases (21.1%) and chronic rejection occurred in 1 case (5.2%).

## Discussion

In this study, we show that vascular reconstruction of segmental intestinal grafts using autologous internal iliac vessels as interpositional grafts greatly facilitates graft implantation and minimizes the risk of vascular complications. Our results indicate that this technique may be useful for transplantation of both intestinal autografts and allografts when the donor terminal SMA and SMV are compromised by a short pedicle and small vessel caliber.

In our earlier attempts, the graft artery and vein were anastomosed to the stump of the recipient's remnant SMA and portal vein. However, in patients with short bowel syndrome, it was often technically difficult or even impossible to accomplish a successful anastomosis due to poor quality or defects in the native SMA [8, 18]. In the setting of IATx, this reconstruction usually put the vascular anastomoses behind the pancreaticojejunostomy or near the proximal enteroenterostomy after closure of the abdominal incision, which puts the anastomosis at great risk of rupture in the event of pancreatic leakage. In fact, one death in our series was directly due to pancreatic leak-related rupture of the SMA anastomosis [15]. Therefore, we prefer to use the aorta distal to the inferior mesenteric artery and vena cava as an anastomotic site, which is relatively farther away from the pancreaticojejunostomy. Benedetti *et al*. [19] reported 12 cases of LR-ITx with a successful direct vascular anastomosis. However, in our experience, we feel that the use of an interpositional graft greatly reduces the tension and decreases the rate of vascular complications in both LR-ITx and IATx. This maneuver extends the short mesenteric vessel pedicle and allows it to reach the infrarenal aorta and vena cava without tension.

Vascular autografts, such as inferior epigastric artery, radical artery, internal mammary artery, inferior mesenteric artery, internal iliac artery, saphenous vein, etc., can be used for *in vitro* vascular reconstruction in living-related liver, kidney, or pancreas transplants [20‒24]. Synthetic vascular grafts have also been used in complex vascular reconstruction, but are associated with a risk of vascular thrombosis and sepsis [25, 26]. In our series, vascular allografts from cadaveric donors were not used for vascular reconstruction because of issues related to donor and recipient histocompatibility and availability. Because of the excellent collateral circulation in the pelvis, ligation of a unilateral internal iliac vessel does not compromise blood supply or venous drainage [27]. In addition, the anatomy of the internal iliac vessels is usually constant with rare variation and they are easy to procure with minimal harm to the recipient. Therefore, the internal iliac artery and vein grafts are suitable vessels that are used for SMA and SMV reconstruction. Similar to our series, Tzakis *et al*. [13] reported three cases of IATx using the internal iliac artery as an interpositional graft with acceptable outcomes. Although the number of patients who will benefit is small, this aggressive procedure allows patients with otherwise unresectable neoplasms to undergo radical resection with negative margins.

During preoperative evaluation, the recipient's iliac arteries should be carefully assessed for the severity of calcification. A heavily calcified iliac artery might cause narrowing of an anastomosis leading to graft dysfunction or loss, which requires use of an alternative vessel, such as the internal mammary artery or inferior mesenteric artery [28]. In addition, caval drainage is theoretically less physiologic than portal drainage, but it usually carries a low risk of dramatic metabolic consequences in the presence of normal liver function [29]. Aside from these limitations, another drawback to this approach is the increase in cold ischemia times during *ex vivo* reconstruction. In our experience, this back-table procedure usually takes 20‒30 min to accomplish.

In summary, our results indicate that *ex vivo* reconstruction of donor terminal SMA and SMV with autologous internal iliac vessels is a safe and effective surgical procedure for intestinal allografts and autografts. This novel procedure reduces the risk of vascular complications and greatly facilitates graft implantation, which may be useful for segmental intestinal transplants when donor mesenteric vessels are of insufficient length or caliber.

## Authors’ Contributions

Study design and concept: G.W., Y.W., and L.B. Acquisition of data: G.W., Y.W., W.M., Z.T., and C.L. Data analysis: Y.W., M.W., and C.L. Manuscript preparation and revisions: G.W., Y.W., W.Z., W.M., and T.L. Study oversight: G.W. All authors read and approved the final manuscript.

## Funding

This work was supported by the grant from the National Natural Science Foundation of China [#81770644] and Key Program of National Natural Science Foundation of Zhejiang Province (LD21H03000).
